# Airway cilia recovery post lung transplantation

**DOI:** 10.1002/iid3.527

**Published:** 2021-09-21

**Authors:** Randy Suryadinata, Kovi Levin, Lynda Holsworth, Miranda Paraskeva, Philip Robinson

**Affiliations:** ^1^ Department of Respiratory and Sleep Medicine,, Victorian Diagnostic Service for PCD The Royal Children's Hospital Melbourne Parkville Victoria Australia; ^2^ Respiratory Diseases Murdoch Children's Research Institute Parkville Victoria Australia; ^3^ Department of Respiratory Medicine, Lung Transplant Service The Alfred Hospital Melbourne Victoria Australia; ^4^ Department of Paediatrics University of Melbourne Parkville Victoria Australia

**Keywords:** cilia, lung transplantation, mucociliary clearance

## Abstract

**Background:**

Normally functioning airway cilia is essential for efficient mucociliary clearance to protect the airway from various insults. Impaired clearance may lead to increased risk of infections and progressive lung damage. Significant morbidity in the immediate post lung transplantation period is associated with airway infection, which we hypothesize may be caused by impaired cilia function.

**Methods:**

Airway cilia beating pattern (CBP) and frequency (CBF) were studied on brushing samples taken from above and below the transplant anastomosis of adult lung transplant recipients (*n* = 20) during routine bronchoscopies at 6, 12, and 26 weeks posttransplant. Bronchoaveolar Lavage (BAL) samples were also collected at each time points.

**Results:**

At 6 weeks posttransplant (*n* = 16), CBP from the donated lung showed reduced beating amplitude with the overall CBF 2.28 Hz slower than the patients' native upper airway cilia (median ± SIQR: 5.36 ± 0.93 Hz vs. 7.64 ± 0.92 Hz, *p* value < .001). At 12 weeks (*n* = 16), donor lungs CBP showed recovery with the difference in CBF reduced to 0.74 Hz (6.36 ± 1.46 Hz vs. 7.10 ± 0.86 Hz, *p* value < .05). Impaired cilia function was not associated with positive BAL cultures.

**Conclusion:**

Reduced cilia function is evident in the first 12 weeks post lung transplant, with both CBP and CBF returning to levels of function indistinguishable to the patients' upper airway cilia beyond this time.

## Graphical abstract

Reduced airway cilia function is evident in the first 12 weeks post lung transplant, with both CBP and CBF returning to levels of function indistinguishable to the patients' upper airway cilia beyond this time. This difference may be a cause for increased risk of airway infection during this period.

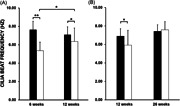

## 1 INTRODUCTION

Lung transplantation is an accepted treatment for various end‐stage lung diseases, with overall survival outcomes improving over the past decade. While this is due, in part, to advances in organ preservation,^1^, ^2^ surgical techniques,^3^ and immunosuppressive regimens,^4^ airway infections in the immediate peri‐operative period remain a common complication, accounting for more than one‐third of deaths in the first year following transplant.^5^

While posttransplant infections are often associated with the use of immunosuppressive agents, lung transplant recipients may be susceptible to respiratory infections from localized pulmonary issues including impaired mucociliary clearance (MCC) of the lungs. In healthy airways, inhaled pollutants and pathogens are trapped in mucus and removed from the airways by coordinated cilia beating. Impaired MCC linked to defective cilia function, as seen in inherited primary ciliary dyskinesia (PCD), results in accumulation of mucus‐trapped harmful particles.^6^ This, in turn, promotes colonization of pathogens and elicits inflammatory responses that may ultimately lead to bronchiectasis. Secondary abnormalities of cilia function present post lung transplant may also lead to impaired MCC, with compromised removal of infectious agents causing further damage.

MCC in the donated lungs has been shown to be impaired following transplantation, with up to 50% loss in clearance observed in some cases.^7^, ^8^ The exact etiology for impaired MCC following lung transplant remains unclear, and early investigations into airway cilia beat frequency analysis (CBF) have yielded contradictory findings.^9^, ^10^, ^11^, ^12^ In a 2012 cross‐sectional study, Thomas et al.^11^ demonstrated comparable cilia beat pattern (CBP) between native cilia and donor lungs in children receiving lung transplantation at random time points between 7 and 12 months posttransplant.^11^ Analysis of CBP in donated lungs following transplantation in adult recipients has not been explored previously. In this current study, we investigated the rate of recovery of CBP and CBF in donated lungs following transplantation in adults at specified time points. We hypothesized that a mismatch between donor and native cilia function may be associated with a higher risk of airway infection.

## 2 PATIENTS AND METHODS

### 2.1 Patients

Twenty adult patients who had undergone either bilateral (*n* = 17) or single (*n* = 3) lung transplantation at the Alfred Hospital between 2018 and 2019 were enrolled (ethics approval no.: HREC/18/Alfred/107). Pediatric lung transplant recipients were excluded, as were out‐of‐state adult recipients. Samplings of airway ciliated epithelial strips were performed using bronchial brushes (Conmed) during routine surveillance bronchoscopies at 6, 12, and 26 weeks posttransplant. At each time point, brush sample of the patient's native cilia was taken from behind the inferior nasal turbinate. Sampling of the donor lung's cilia was taken from below the anastomosis on the apical segment of the right lower lobe. For the two patients who received single left lung transplantation, brushings were taken from the left lingula lobe. Brushing samples were then placed in Medium‐199 pH 7.6 (Sigma‐Aldrich) supplemented with 200 units/ml penicillin, 200 µg/ml streptomycin, and 0.5 µg/ml amphotericin B solution (Sigma‐Aldrich). Ischemic time was defined as the time from cessation of perfusion to re‐perfusion of donor lungs.

### 2.2 High‐speed video microscopy

Assessment of CBP on epithelial strips was performed within 4 h of collection, as described previously.^13^ Briefly, the epithelial strips were loosened from the brush by agitation. Small portion of the cell suspension was then mounted on a chambered glass slide and placed on 35°C heating stage positioned on a Nikon Eclipse 80i microscope. Cilia beating were examined on various planes using a Plan Apochromat ×100 objective—oil immersion lens (Nikon) and recorded on FastCam SA3 video camera (Photron) at a rate of 500 frames‐per‐second (fps). Detailed analyses were performed by playing the recorded data at a reduced 30 fps rate.

CBF analysis was calculated by dividing the recording rate by the number of frames required to complete 10 beating cycle, multiple by 10 (i.e., CBF = (500/no. of frames for 10 beat cycles) × 10). For accuracy, 10 recordings of ciliated epithelial strips from different areas of each sample were made and the average CBF calculated.

### 2.3 Statistics

Data were presented as median ± semi‐interquartile range (SIQR) for CBF of both native and donor lungs' cilia. One‐tailed Mann–Whitney test was used to compare the difference in the airways CBF at different time points following transplantation. Two‐tailed Spearman's correlation analysis was used to demonstrate the relationship between organ ischemic time or postoperative ventilation time and donor lungs' CBF at 6 weeks posttransplant. *p* values less than .05 indicated a statistically significant difference between comparisons.

## 3 RESULTS

### 3.1 Demographics

The study cohort comprised of 16 males and four females with an average age of 59 years (range: 28–72 years). The spectrum of lung transplants performed, and underlying lung diseases of the subjects are summarized in Table 1. During the study period, six patients missed their bronchoscopy appointment either at 12 weeks or 26 weeks due to personal, medical, or logistical reasons (including cessation of routine bronchoscopy during coronavirus disease 2019 pandemic). Additionally, the upper airway epithelial strips of one patient were found denuded of cilia during 6‐week bronchoscopy procedure and no data were therefore available for this time‐point in the final analysis. As such, a complete 6 weeks bronchoscopy data set were collected from 19 patients, while complete data from 12 week and 26 week bronchoscopies was obtained from 17 patients. Comparative CBF analysis between 6‐ and 12 weeks posttransplant was performed on 16 patients, while 12 week‐to‐6 month comparison study was done on 14 patients. Correlation study between donor lungs' CBF at 6 weeks posttransplant and postoperative intubation time in intensive care unit (ICU) was performed on 17 patients, as three patients required further noninvasive ventilation (NIV) after initially extubated (for 2.5, 9, or 15 h). Correlation between donor lungs' CBF at 6 weeks posttransplant and ischemic time was available for 19 patients, as the donor lungs for one patient were transplanted following ex vivo lung perfusion.

**Table 1:** Patients' demographics (*n* = 20)

Average age	59 (28–72)
Gender, male	16
Types of lung transplant	
BSLTx	17
Left SLTx	2
Right SLTx	1
Respiratory diseases	
IIP	8
*a. "Unclassifiable" ILD*	*4*
*b. IPF*	*2*
*c. NSIP*	*1*
*d. Familial pulmonary fibrosis*	*1*
COPD	6
CTD‐ILD	2
CF	2
Sarcoid	1
CLAD	1

Abbreviations: BSLTx, bilateral sequential lung transplant; CF, cystic fibrosis; CLAD, chronic lung allograft dysfunction COPD, chronic obstructive pulmonary disease; CTD‐ILD, connective tissue associated ILD; IIP, idiopathic interstitial pneumonia; ILD, interstitial lung disease; IPF, idiopathic pulmonary fibrosis; NSIP, nonspecific interstitial pneumonitis; SLTx, single lung transplant.

As part of routine post‐transplant protocol, bronchial alveolar lavage (BAL) samples were taken during each bronchoscopy procedure for microbiological surveillance (Table 2). Airway lavage was always conducted after cilia brush samples had been obtained. Six patients were culture positive for *Pseudomonas aeruginosa, Klebsiella pneumoniae* or MSSA at 6 weeks posttransplant, with five of these patients culture‐negative by the subsequent bronchoscopy at 12 weeks after targeted antimicrobial therapy. One patient tested positive for *P. aeruginosa* and *Aspergillus. fumigatus* at 12 weeks posttransplant, having been clear previously. Both organisms were absent at lavage at the 6‐month bronchoscopy. One additional patient returned a positive culture for *Aspergillus* spp. during their 6‐month bronchoscopy, which was not detected on earlier lavages.

**Table 2:** Distribution of microbial detected during routine bronchoscopy and their relationship with donor lungs' cilia function

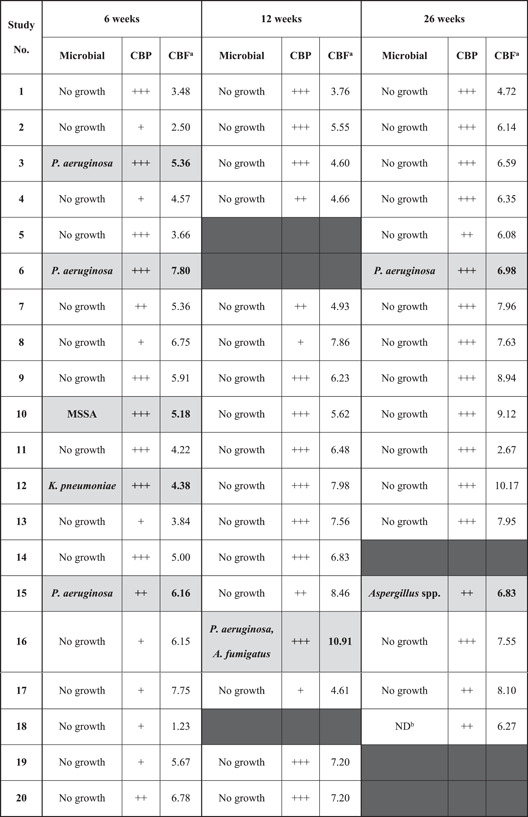

*Note*: Samples with positive for microbial growth are highlighted in gray, where *A. fumigatus, Aspergillus fumigatus; Aspergillus* spp., unidentifiable *Aspergillus* species; *P. aeruginosa, Pseudomonas aeruginosa; K. pneumoniae, Klebsiella pneumoniae*; MSSA, methicillin‐sensitive *Staphylococcus aureus*.

CBP is described according to the proportion of normal to defective beating observed under high‐speed video microscopy, with **+++**: mostly normal beating; **++**: mix of normal and defective beating; **+**: mostly defective beating.

^a^CBF is represented as the average of calculated CBF from each sample.

^b^ND, BAL cultures not done.

Columns where sampling was unable to be performed are blacked out.

### 3.2 Cilia function recovery posttransplant

The overall analysis of ciliary beat pattern (CBP) assessed in donor lungs following transplantation is summarized in Table 2. Normal CBP displayed wave pattern with a range of motion (amplitude) that comprise of a strong forward stroke that is followed by a recovery bending stroke. Defective beating is described as CBP that is reduced in amplitude. The majority of the epithelial strips obtained from the donor lungs during 6‐week bronchoscopy procedure appeared unhealthy with damaged membranes despite being well ciliated. More than half of the samples collected from the donor lungs displayed defective cilia beating. In comparison, the brush samples collected from the donor lungs at 12 weeks bronchoscopy appointment showed healthier epithelial strips with a large number of the samples collectively showing normal CBP. More efficient clearance of cell debris was also evident at 12 weeks posttransplant. At 26 weeks posttransplant, the donor lungs' CBP of most patients had returned to normal. One patient with CF showed minimal improvement in CBP throughout the study period.

**Figure 1:**
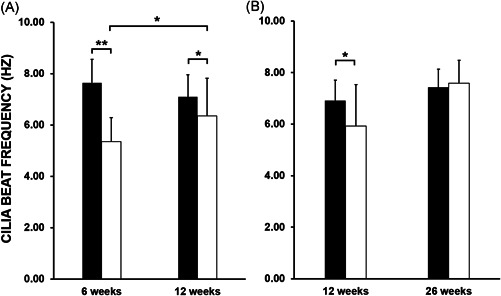
Cilia beat frequency analysis (CBF) following lung transplantation. Median ± SIQR CBF of donor lungs' cilia (white bars) and patients' native cilia (black bars) were compared. (A) Donor lungs' CBF was significantly slower than the patients' native CBF at 6 weeks posttransplant. The CBF of the donor lungs showed significant improvement at 12 weeks posttransplant (*n* = 16). (B) Continuing improvement in CBF was observed in the donor lungs at 26 weeks posttransplant, with minimal difference in CBF between the donor lungs' cilia and patients' native cilia recorded (*n* = 14). Statistical significance of *p* value < .05 (*) and *p* value < .001 (**) were demonstrated

The median CBF of the donor lungs at 6 weeks posttransplant was 2.28 Hz slower compared with the patients' native cilia (5.36 ± 0.93 Hz vs. 7.64 ± 0.92 Hz, *p* value < .001). At 12 weeks posttransplant, donor lungs' cilia showed recovery with the overall difference in CBF reduced to 0.74 Hz (6.36 ± 1.46 Hz vs. 7.10 ± 0.86 Hz, *p* value < .05). There was a statistically significant difference in donor lungs' CBF from the 6 week to 12 week study (5.36 ± 0.93 Hz vs. 6.36 ± 1.46 Hz, *p* value < .05) (Figure 1A). Donor lungs' CBF showed a further improvement at 26 weeks posttransplant compared with 12 weeks prior, with the calculated difference recorded at 1.67 Hz (7.59 ± 0.88 Hz vs. 5.93 Hz ± 1.60 Hz, *p* value > .05). Analysis of donor lungs' CBF at 26 weeks posttransplant also indicated full recovery of cilia function when compared with patients' native cilia, with a nonsignificant difference (7.59 ± 0.88 Hz vs. 7.42 ± 0.71 Hz, *p* value > .05) (Figure 1B). For reference, our PCD diagnostic service has recorded a similar median CBF values from nasal brushing samples obtained from non‐PCD patients. Our record is in agreement with a recent study demonstrating a median upper airway's CBF of 8.00 Hz (range 6.50–9.80 Hz) when recorded at 37°C, with slower median CBF value observed at lower temperatures.^14^ A further comparison between BAL culture results and cilia function revealed that the presence of bacteria or fungi in the lavages did not affect both CBP and CBF of the donor lungs, as patients with positive microbiological cultures maintained normal cilia beating function (Table 2).

**Figure 2:**
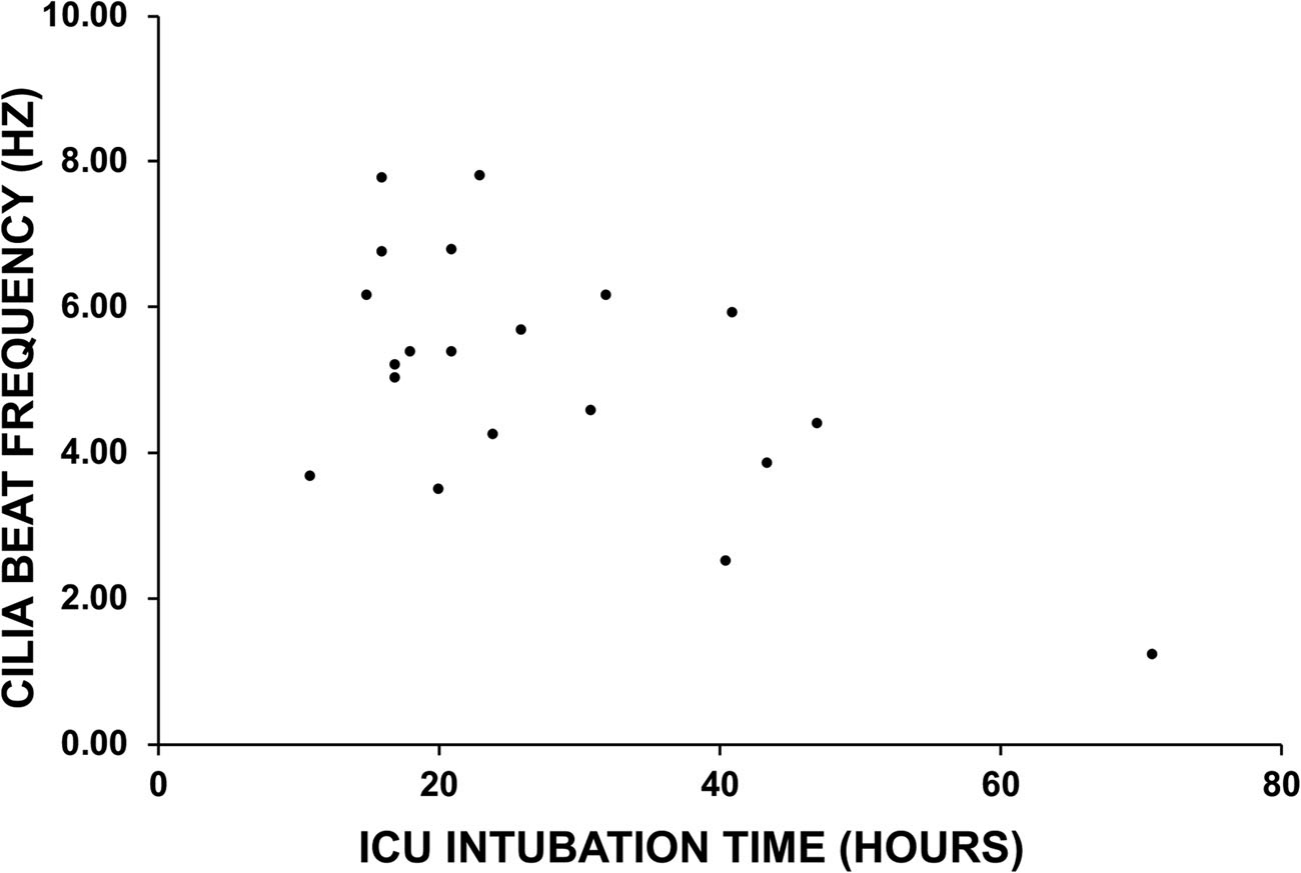
Relationship between intensive care unit (ICU) intubation time and donor lungs' cilia function following transplantation. A Spearman's correlation analysis revealed a weak negative correlation (*R* = −0.4957) between time spent in ICU for postoperative intubation and the associated donor lungs' CBF assessed at 6 weeks posttransplant (*n* = 17). CBF, cilia beat frequency

The relationship between donor lungs' cilia function and postoperative intubation time was investigated in 17 patients. The median for postoperative intubation time was 21 h (range: 15–71 h). There was a weak negative correlation (*R* = −0.4957, *p* value < .05) between the length of intubation time required in ICU and donor lungs' CBF assessed at 6‐week bronchoscopy procedure (Figure 2), with patients requiring shorter intubation times showing moderately faster cilia beating in their donor lungs. The patient with the longest intubation time (71 h) demonstrated the lowest airway CBF (1.23 Hz). As cilia function recovered at the later time‐points, the correlation between postoperative intubation time and donor lungs' CBF was no longer observed (data not shown).

**Figure 3:**
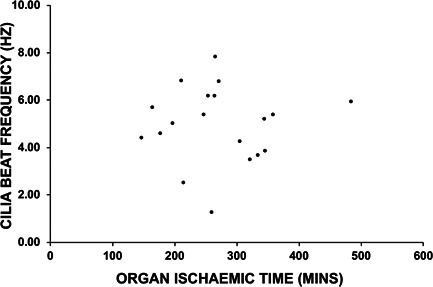
Correlation analysis between organ ischemic time and donor lungs' cilia function post‐transplant. A Spearman's correlation analysis revealed no association (*R* = −0.043) between ischemic time and donor lungs' CBF assessed at 6 weeks posttransplant (*n* = 19). CBF, cilia beat frequency

The relationship between the donor lungs' CBF and organ ischemic time was also studied (*n* = 19). The median ischemic time in this study was 265 min (range: 148–485 min), and analysis revealed no correlations between ischemic time and donor lungs' CBF assessed at the 6‐week (Figure 3), 12‐week or 26 week airway sampling (data not shown).

## 4 DISCUSSION

This present study has described a period of impaired airway cilia function and by extension MCC in the immediate post lung transplant period that in most case is not fully resolved until 12 weeks posttransplant. In healthy lungs, airway cilia play a critical role for ensuring effective MCC. Previous investigations on cilia function in donor lungs posttransplant have yielded conflicting results. In one study examining single lung transplant recipients, a significant reduction in CBF in the donor lung compared with that of the native lung following transplantation was described.^12^ In contrast, similar studies have reported no difference in donor lungs' CBF posttransplant.^9^, ^10^, ^11^ The findings from these early studies were limited by sampling protocol where ciliated epithelial samples were taken at random times over a large study period gap, with the possibility that a proportion of samples were taken at a later stage post‐transplant. Our study is, to our knowledge, the first to examine CBF and CBP at multiple, pre‐determined time points posttransplant as well as studying these parameters of MCC at the relatively early stage of 6 weeks posttransplant. While it is difficult to make direct comparisons between our findings and those from previous studies due to variability in patients' demographics and the techniques used for assessing cilia function, our findings would suggest that the lack of any difference in CBF observed in earlier studies, is likely due to majority of the donor lungs' cilia function having recovered at the time of testing.

Interestingly, our study has recorded similar CBF values between donated lungs and the patients' upper airway cilia at 26 weeks posttransplant. This finding differs to the notion that lower airways' cilia beat slower than the cilia isolated from the upper airways but is in agreement with early studies which observed similar CBF values from the nasal, tracheal, and bronchial epithelial cells.^15^, ^16^

The basis for cilia dysfunction in donor lungs following transplantation is unclear. From this small sample, positive BAL culture for either bacteria or fungi appeared to play no role in cilia dysfunction. Viral infections have been previously implicated to cause ciliary dyskinesia and cilia loss,^17^, ^18^, ^19^ however, the airway lavages are not routinely tested for respiratory viruses, and we are unable to comment on this. Organ ischemic time was also not associated with impaired donor lungs' CBF. Interestingly, our data suggested a weak correlation between donor lungs' CBF and postoperative intubation time. Previous studies in nontransplant situations have demonstrated that prolonged exposure to positive pressure ventilation significantly reduces tracheal CBF and overall MCC.^20^, ^21^ Similarly, exposures to high normobaric oxygen concentration lead to decreases in CBF and overall impairment in fluid flow and cellular damage to mammalian tracheal epithelium.^22^, ^23^

A possible relationship between postoperative intubation time and cilia function is intriguing, however, the data generated from the current study is limited and requires more detailed investigations. In addition to the small sample size, the patients recruited for this study represent individuals who spent a relatively short time intubated, and this initial comparison also did not account for further postsurgical complications. For example, three patients in our study required NIV for 2, 9, or 15 h after initially extubated for 15, 9, or 2.5 h, respectively. Whether additional ventilation support exacerbates cellular damage that may further compromise cilia function in donor lungs remains to be investigated and would certainly provide better insights into understanding the role of postsurgical ventilations in cilia dysfunction.

Other aspects of the lung transplant process which have been associated with cilia dysfunction in donor lungs also require consideration in interpreting our results. Commonly used immunosuppressive therapy regimes, including tacrolimus, mycophenolate sodium, and prednisone or cyclosporine, azathioprine, and prednisone, have both been shown to result in reductions in both CBF and overall MCC in rats.^24^

The impairment of cilia function at 6 weeks post‐transplant suggests a period of impaired mucociliary clearance and possible increased risk of lung function and damage (exacerbated by the concomitant administration of immunosuppressive therapy) that may be minimized by interventions that could potentially optimize mucociliary clearance in that period. Interventive trials of agents which promote mucociliary clearance such as nebulized hypertonic saline or dry powder mannitol, in addition to enhanced chest physiotherapy regimes, may minimize the degree of any lung damage that may occur during this period and possibly be reflected as better lung health long term.

In conclusion, our present study has shown that cilia dysfunction in donor lungs following transplantation is significant at 6 weeks posttransplant and shows recovery at 12‐weeks with often further improvement at later testing times. Further study is required to assess the prevalence of airway infection and cilia function in adult patients during the first few months post lung transplantation.

## CONFLICT OF INTERESTS

The authors declare that there are no conflict of interests.

## AUTHOR CONTRIBUTIONS

*Conceptualization*: Miranda Paraskeva and Philip Robinson. *Data curation*: Randy Suryadinata, Kovi Levin, and Lynda Holsworth. *Formal analysis*: Randy Suryadinata, Kovi Levin, and Philip Robinson. *Funding acquisition*: Philip Robinson. *Investigation*: Randy Suryadinata. *Methodology*: Randy Suryadinata, Kovi Levin, Lynda Holsworth, Miranda Paraskeva, and Philip Robinson. *Project administration*: Kovi Levin and Lynda Holsworth. *Writing—original draft*: Randy Suryadinata. *Writing—review and editing*: Randy Suryadinata, Kovi Levin, Lynda Holsworth, Miranda Paraskeva, and Philip Robinson.
